# Identification of a Candidate *restorer-of-fertility* Gene *Rf3* Encoding a Pentatricopeptide Repeat Protein for the Cytoplasmic Male Sterility in Soybean

**DOI:** 10.3390/ijms23105388

**Published:** 2022-05-11

**Authors:** Yanyan Sun, Yan Zhang, Shungeng Jia, Chunjing Lin, Jingyong Zhang, Hao Yan, Bao Peng, Limei Zhao, Wei Zhang, Chunbao Zhang

**Affiliations:** 1 Soybean Research Institute, Jilin Academy of Agricultural Sciences, Changchun 130033, China; sunyy@cjaas.com (Y.S.); zhangyanxii@hotmail.com (Y.Z.); jiashungeng@hotmail.com (S.J.); lincj@cjaas.com (C.L.); zhangjy@cjaas.com (J.Z.); yanhao@cjaas.com (H.Y.); pb@cjaas.com (B.P.); lmzhao@cjaas.com (L.Z.); zw0431@cjaas.com (W.Z.); 2 Key Laboratory of Hybrid Soybean Breeding of the Ministry of Agriculture and Rural Affairs, Changchun 130033, China

**Keywords:** soybean, cytoplasmic male sterility, restorer line, restorer-of-fertility gene, fine mapping

## Abstract

The cytoplasmic male sterility/restorer-of-fertility (CMS/*Rf*) system plays a vital role in high-efficiency hybrid seed production in crops, including soybean (*Glycine max* (L.) Merr.). The markers linked to fertility restoration and the *restorer-of-fertility* (*Rf*) genes are essential because they can facilitate the breeding of new CMS lines and production of commercial hybrid soybean seeds. To date, several soybean *Rf* genes have been mapped to various genetic loci in diverse genetic populations. However, the mapping range of restorer genes remains narrow, with relatively limited practical applicability. Therefore, in the present study, F_2_ and F_3_ segregating populations derived from the CMS line JLCMS5A crossed with the restorer line JLR2 were developed and used for *Rf3* gene fine mapping. Genetic investigation indicated that the restorer line JLR2 was controlled by a single dominant gene, *Rf3*. By integrating bulk-segregant analysis and next-generation sequencing, a 4 Mb region on chromosome 9 was identified, which was most likely the target region harboring the candidate gene responsible for fertility restoration. This region was further narrowed down to 86.44 Kb via fine mapping in F_2_ and F_3_ populations using SSR, InDel, and dCAPS markers. This region contained 10 putative genes (*Glyma.09G171100*–*Glyma.09G172000*). Finally, *Glyma.09G171200*, which encodes a mitochondria-targeted pentatricopeptide repeat protein, was proposed as the potential candidate for *Rf3* using sequence alignment and expression analysis in restorer and CMS lines. Based on single-nucleotide polymorphisms in *Glyma.09G171200*, a CAPS marker co-segregated with *Rf3* named CAPS1712 was developed. Our results will be fundamental in the assisted selection and creation of potent lines for the production and rapid selection of novel restorer lines.

## 1. Introduction

Soybean (*Glycine max* (L.) Merr.), which originated in China and has been cultivated for over 5000 years, is a great source of quality protein and vegetable oil. In recent years, the demand for soybean has grown rapidly, but the supply remains inadequate. Hybrid breeding is an important approach to increase the yield and improve the quality of crops, and it has been successfully applied in rice, maize, wheat, rapeseed, and other crops. However, a high degree of self-pollination and availability of limited germplasm resources restrict the use and spread of commercial hybrid cultivars.

Cytoplasmic male sterility (CMS) is the maternally inherited inability to produce functional pollen, which is widespread in vascular plants, being recorded in nearly 200 species worldwide [1]. Fertility loss in CMS plants is often attributed to unusual chimeric open reading frames (ORFs) in the mitochondrial genome [2], and it can be reversed by the *restorer-of-fertility* (*Rf*) genes located in the nuclear genome [3]. The restoration of CMS via *Rf* genes can be achieved at the genomic, transcriptomic, proteomic, and metabolic levels [4]. To date, the CMS/*Rf* systems have been widely identified and applied to improve the yield of many crops, such as rice [5], maize [6], wheat [7], pepper [8], and Brassicaceae crops [9]. Therefore, the use of a CMS/*Rf* system is an ideal strategy for large-scale hybrid soybean production.

To date, *Rf* genes have been successfully isolated from several plants, including *Rf2* and *Rf4* in maize [6,10]; *Rf-PPR592* in petunia [11]; *Rfo* in radish [12]; *Rf1, Rf2, Rf4, Rf5, Rf6*, and *Rf17* in rice [1,13,14,15,16,17]; *Rf1* in sugar beet [18]; *Rfp*, *Rfn*, and *Rfh* in rapeseed [19,20,21]; and *Rf1* and *Rf3* in wheat [7], among others. Most of these *Rf* genes encode pentatricopeptide repeat (PPR) proteins localized in the mitochondria. In addition, several other types of *Rf* genes encode non-PPR proteins. For instance, *Rf2* and *Rf4* in maize encode an aldehyde dehydrogenase [10] and a basic helix–loop–helix (bHLH) transcription factor [6], respectively. Moreover, *Rf2* and *Rf17* in rice encode a glycine-rich protein [14] and an acyl-carrier protein synthase [17], respectively. Finally, *Rf1* in sugar beet encodes a peptidase [18].

In soybean, only *GmPPR576* has been identified on chromosome 16 between the markers BARCSOYSSR_16_1067 and BARCSOYSSR_16_1078, and it is considered the restorer-of-fertility gene of the NJCMS1A line [22]. According to Wang et al. (2021), *GmPPR576*, which encodes a PPR protein, belongs to the restorer-of-fertility-like (RFL) *Rf* gene family and is targeted to the mitochondria. In addition, several soybean *Rf* genes have been mapped to various genetic loci in different genetic populations, and most of these genes are located on chromosome 16. For instance, Zhao et al. (2007) and Wang et al. (2010) reported that in the restorer line JIHUI 1, the genetic distances between the *Rf* gene and the SSR markers Sctt011 and Satt547 were, respectively, 3.6 and 5.4 cM [23,24]. Meanwhile, Dong et al. (2012) suggested that in the restorer line FuHui9, the *Rf* gene was mapped on chromosome 16, between the markers BARCSOYSSR_16_1064 and BARCSOYSSR_16_1082, at the distances of 0.59 and 0.83 cM, respectively [25]. Wang et al. (2016) showed that the M-type CMS line restorer-of-fertility gene *Rf-m* is located within a 162.4 Kb region on chromosome 16 and is flanked on each side by markers GmSSR1602 and GmSSR1610 at a distance of 0.11 and 0.25 cM, respectively [26]. *Rf* genes have also been found on other chromosomes. For instance, Yang et al. (2007) used N8855 sterile and restorer lines as materials and selected two polymorphic markers linked to *Rf* genes, which are located on chromosomes 5 and 7 [27]. However, the mapping range of most *Rf* genes in soybean is limited, and the underlying molecular mechanisms remain unknown, thus restricting the development of commercial varieties.

In this context, the identification and characterization of *Rf* genes is an effective and important approach to rapidly establish a CMS/*Rf* system for commercial application in hybrid soybean production. Therefore, in the present study, *Rf3* in soybean was physically mapped using published and newly developed markers. Specifically, an 86.44 Kb candidate interval on chromosome 9 was identified by integrating bulk-segregant analysis and next-generation sequencing (BSA-Seq) using F_2_ and F_3_ segregation populations. In this region, *Glyma.09G171200*—a PPR family gene localized in the mitochondria—was considered a candidate for *Rf3* based on sequence alignment and expression analysis. Our findings will serve as a reference in the assisted selection and development of potent lines in the production and rapid selection of novel restorer lines. Our results will lay a theoretical foundation for exploring the mechanisms underlying the CMS/*Rf* system in soybean.

## 2. Results

### 2.1. Gametophyte Sterility in the Soybean CMS Line JLCMS5A

**Figure 1:**
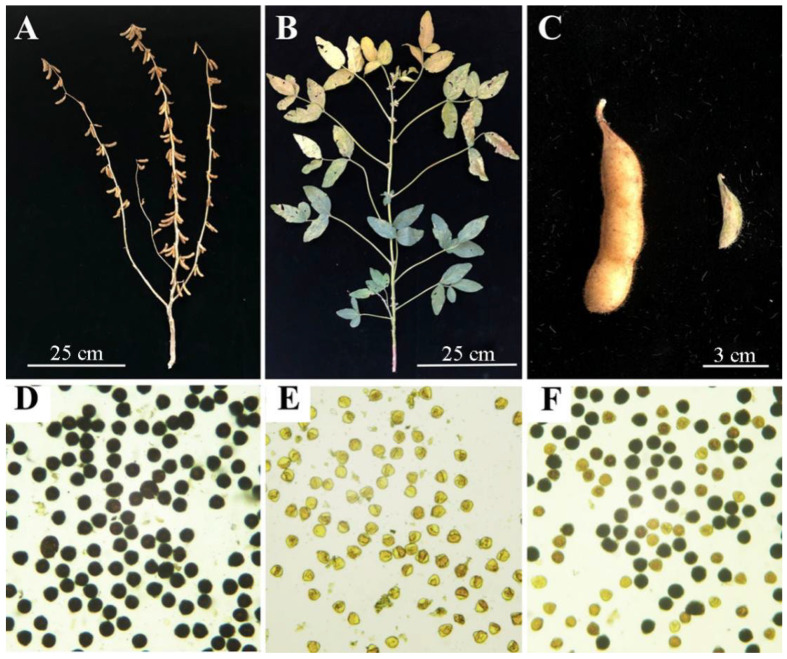
Phenotypic and cytological characterization of the restorer and cytoplasmic male sterile (CMS) lines. (**A**) Restorer line JLR2. Scale bar: 25 cm. (**B**) CMS line JLCMS5A. Scale bar: 25 cm. (**C**) Pods of JLR2 (left) and JLCMS5A (right). Scale bar: 3 cm. (**D**–**F**) Mature pollen grains of JLR2, JLCMS5A, and F_1_ plants stained with I_2_-KI.

JLR2 is a restorer line of the soybean CMS line JLCMS5A. JLCMS5A was indistinguishable from JLR2 during vegetative growth, except that JLCMS5A produced small and fleshy seedless pods after flowering (Figure 1A–C). JLR2, JLCMS5A, F_1_, and F_2_ generation pollen were subjected to the I_2_-KI test. Microscopic observations revealed that all pollens produced by the restorer line JLR2 were viable (Figure 1D). Conversely, the CMS line JLCMS5A produced only non-viable pollen grains (Figure 1E). The F_1_ hybrids were semi-fertile, with only half of the pollen being viable (Figure 1F). The I_2_–KI test was also performed on the F_2_ generation pollen of JLCMS5A × JLR2, and the pollen fertility of 217 F_2_ plants was investigated. There were 121 fertile pollen plants and 96 pollen semi-sterile plants, meeting the 1:1 ratio according to the chi-square test [χ^2^ = 1.44 < χ^2^_(0.05,1)_ = 3.84]. Based on the pollen fertility segregation ratios of F_2_ plants, we confirmed that JLCMS5A is gametophyte sterile. Thus, the fertility restoration of JLCMS5A by JLR2 is a qualitative trait controlled by a pair of dominant genes, which were named *Rf3*.

### 2.2. BSA-Seq

Based on microscopic observations, DNA bulks of two parent lines (male and female) as well as fertile (F) and semi-fertile (SF) plants from the F_2_ segregation population were prepared and subjected to BSA-Seq. From the sequencing analysis, 85.10 Gb of raw data were obtained; the average Q20, Q30, and GC content values were 97.22%, 92.40%, and 36.76%, respectively. After filtering out reads containing unknown bases (N), low-quality data, and only adaptor reads, a total of 12,707,252,100 bp clean reads were obtained from the male sample, with the Q20 value of 96.59%, Q30 value of 91.16%, and GC content of 36.80%. Meanwhile, 10,205,530,500 bp clean reads were obtained from the female sample, with the Q20 value of 97.39%, Q30 value of 92.68%, and GC content of 37.99%. Finally, 31,090,225,200 and 30,918,562,800 bp clean reads were obtained from the F and SF samples, with the Q20 values of 97.72% and 97.18%, Q30 values of 93.54% and 92.22%, and GC content of 36.14% and 36.10%, respectively. We then mapped these clean reads to the reference genome, which allowed for the mapping of 99.48% reads on average. The sequencing depths for the four samples ranged from 8.64× to 24.59×, with an average depth of 17.09×, and 96.22% of the genome had at least 1× coverage (Table 1). As a result, all samples have enough data, qualified sequences, normal GC distribution, and normal comparison results, and the above data can be used for subsequent variation detection and correlation analysis.

**Figure 2:**
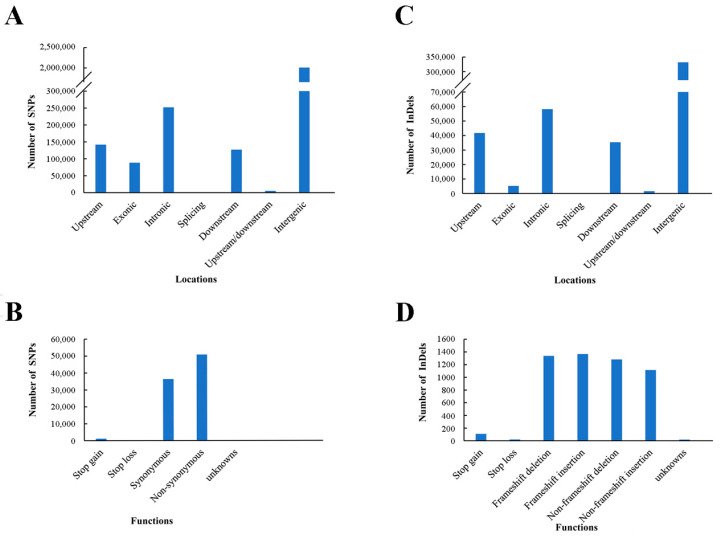
Statistics of the genomic loci of single-nucleotide polymorphisms (SNP) and InDel and their functional annotation. (**A**) Statistics of SNP genomic loci. (**B**) Statistics of SNP functional annotation. (**C**) Statistics of InDel genomic loci. (**D**) Statistics of InDel functional annotation.

To conduct analyses of SNPs and InDels, SNPs and InDels were first called between DNA bulks and the Zhonghong13 reference genome using GATK3.8. A total of 2,627,469 SNPs and 474,499 InDels were identified between the SF and F bulks. There were significantly more SNPs than InDels. Most of the SNPs were in the intergenic region (*n* = 2,011,863), followed by intronic (*n* = 252,156), upstream (*n* = 141,803), downstream (*n* = 127,175), exonic (*n* = 88,602), upstream/downstream (*n* = 5344), and splicing (*n* = 526) regions (Figure 2A). Moreover, some SNPs were predicted to produce non-synonymous (*n* = 50,820), synonymous (*n* = 36,335), stop–gain (*n* = 1175), and stop–loss (*n* = 270) effects, giving rise to phenotypic variations (Figure 2B).

Furthermore, of the 474,499 InDels, over 70% were in the intergenic region (*n* = 332,160), followed by the intronic (*n* = 58,203), upstream (*n* = 41,715), downstream (*n* = 35,367), exonic (*n* = 5262), upstream/downstream (*n* = 1598), and splicing (*n* = 194) regions (Figure 2C). According to the functional annotation, the InDels variations included frameshift insertions (*n* = 1367), frameshift deletions (*n* = 1339), non-frameshift deletions (*n* = 1282), non-frameshift insertions (*n* = 1116), stop–gain variations (*n* = 109), and stop–loss variations (*n* = 22), which may lead to many types of phenotypic changes, including fertility transition (Figure 2D). SNPs and InDels were retained for rapid genetic mapping.

### 2.3. Fine Mapping of Rf3 Gene in JLR2

According to the result of BSA-Seq, we screened for the markers of homozygous differences between the two parents. However, these markers neither possessed more than two alleles nor showed any polymorphism between the F and FS bulks. Following quality control, only 1,557,956 polymorphic marker loci (SNPs and InDels) were used for the calculation of the All index (SNPs and InDels). Then, the ΔAll-index values for all sites and windows were obtained by calculating the difference in the All-index values between the two offsprings, and a Manhattan diagram of the ΔAll-index values was drawn. A candidate region significantly associated with male sterility traits in the range of 38–42 Mb on chromosome 9 was identified based on the ΔAll index, suggesting that *Rf3* may be located in this range. To date, no other published *Rf* gene of soybean has been reported in this range, indicating that *Rf3* is a novel restorer-of-fertility gene.

**Figure 3:**
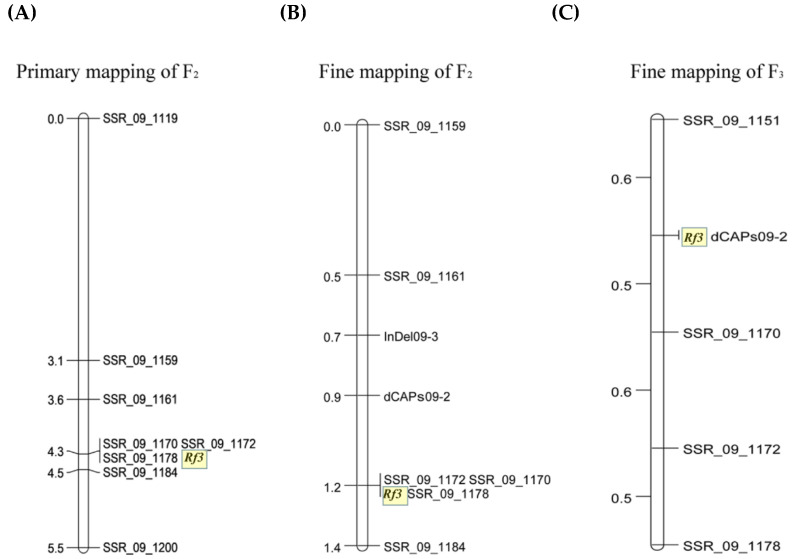
(**A**–**C**) Genetic mapping of the *Rf3* locus on chromosome 9.

To further narrow the *Rf3* locus, 85 pairs of SSR markers on chromosome 9 of soybean were used for BSA. Sixteen SSR markers showed polymorphism between the two parents, as well as between the two F_2_ bulked samples. Of these, eight polymorphic SSR markers were used to analyze 217 individual plants in the F_2_ population and construct a genetic map of the *Rf3* gene (Figure 3A). To narrow down the region harboring *Rf3*, eight pairs of InDel markers and four pairs of dCAPS markers were designed between SSR_09_1161 and SSR_09_1170. Among these, one InDel (InDel09-3) and one dCAPS marker (dCAPs09-2) were polymorphic. We evaluated F_2_ and F_3_ plants using these 10 markers and calculated their genetic distances. These results revealed that *Rf3* is located in the interval between dCAPs09-2 and SSR_09_1170 (Figure 3B,C).

### 2.4. Rf3 Gene Location

**Figure 4:**
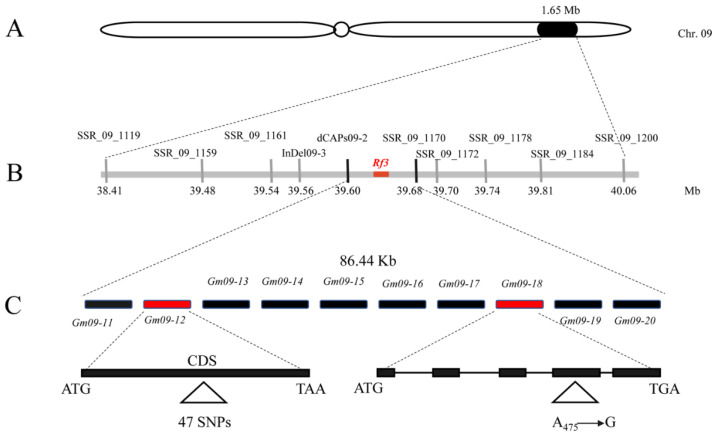
Physical mapping of the *Rf3* locus. (**A**) The *Rf3* locus is located on chromosome 9 according to BSA-Seq and primary mapping. (**B**) Fine mapping of the *Rf3* region on chromosome 9. The *Rf3* locus is mapped to an 86.44 Kb region between the markers dCAPs09-2 and SSR_09_1170. (**C**) The 86.44 Kb region containing 10 putative genes according to the Williams 82 reference genome. *Gm09-11–Gm09-20* indicate *Glyma.09G171100–Glyma.09G172000*, respectively.

According to the physical map of the reference genome of Williams 82, the locations of the markers dCAPs09-2 and SSR_09_1170 are at 39.60 and 39.68 Mb, respectively (Figure 4A,B), at a physical distance of 86.44 Kb. This 86.44 Kb region harbors 10 putative genes (*Glyma.09G171100*–*Glyma.09G172000*) (Figure 4C).

Subsequently, the genomic sequences of 10 genes in this region were cloned from the CMS line JLCMS5A [S (*rf3rf3*)] and the restorer line JLR2 [N (*Rf3Rf3*)]. Following sequence alignments, we found only two genes (*Glyma.09G171200* and *Glyma.09G171800*) that differed between JLCMS5A and JLR2 (Figure 4C). *Glyma.09G171200* harbored 47 SNPs, leading to 31 amino acid variations (Tables S1 and S2). However, most of the mutated amino acids were concentrated on the right subunit of the Glyma.09G171200 protein, and these mutations also altered the interactions between amino acids (Figure S1). *Glyma.09G171800* contained two transitions: A to G at 294 bp and G to A at 475 bp. The last transition led to amino acid variation (K to E).

To confirm whether the SNPs in these two genes are related to fertility restoration, we cloned the two genes from the restorer and CMS lines of another soybean cultivar and performed genome sequence alignment. In these restorer and CMS lines, the CDS of *Glyma.09G171200* contained SNPs, but the sequence was normal in all restorer cultivars and Williams 82 (the above restorer line and Williams 82 have been proved to have fertility recovery ability by experiments on test cross) (Figure S2). In the CMS lines with the same background, *Glyma.09G171200* possessed SNPs at the same positions (Figure S2). However, the SNP at the 294 bp position in the CDS of *Glyma.09G171800* was consistent with that in the CMS lines and Williams 82. Conversely, at the 475 bp site, the sequence was normal in all restorer cultivars and Williams 82 (Figure S3). Regarding function, only one annotated gene (*Glyma.09G171200*) belongs to the PPR protein family (information on the 10 genes is presented in Table 2). Based on these results, we concluded that *Glyma.09G171200* may be the *Rf3* gene in JLR2.

### 2.5. Restorer-of-Fertility Molecular Markers

**Figure 5:**
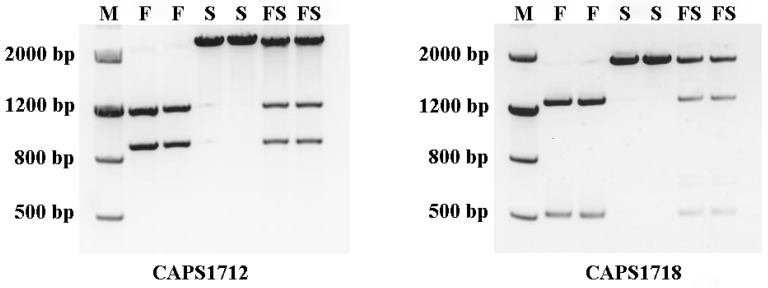
The CAPS markers of *Glyma.09G171200* and *Glyma.09G171800* co-segregated with *Rf3*. M, marker; F, restorer line; S, cytoplasmic male sterile line; FS, semi-fertile F_2_ plants.

Based on the SNPs of *Glyma.09G171200* and *Glyma.09G171800*, CAPS markers named CAPS1712 and CAPS1718 were developed. These two markers are useful for predicting the phenotypes of the offspring of JLCMS5A×JLR2. After amplification and digestion by Sca I restriction endonuclease, the CAPS1712 marker produced different bands in fertile, sterile, and semi-fertile plants. Two fragments, located between the 800 and 1200 bp loci, were considered homozygous at the restorer-of-fertility gene locus (*Rf3Rf3*), indicating fertile plants. A single band greater than 2000 bp was considered homozygous but lacked the restorer-of-fertility gene allele (*rf3rf3*), indicating sterile plants. Three fragments were considered heterozygous at the restorer-of-fertility gene locus (*Rf3rf3*), indicating semi-fertile plants (Figure 5).

For CAPS1718, after amplification and digestion by BspEI restriction endonuclease, the CAPS1718 marker also produced different bands in fertile, sterile, and semi-fertile plants. Two fragments, one located between the 500 and 800 bp loci and the other located between the 1200 and 2000 bp loci, were considered homozygous at the restorer-of-fertility gene locus (*Rf3Rf3*), indicating fertile plants. A single band on 2000 bp loci was considered homozygous but lacked the restorer-of-fertility gene allele (*rf3rf3*), indicating sterile plants. Three fragments were considered heterozygous at the restorer-of-fertility gene locus (*Rf3rf3*), indicating semi-fertile plants (Figure 5).

### 2.6. Expression and Localization of Candidate Rf3

**Figure 6:**
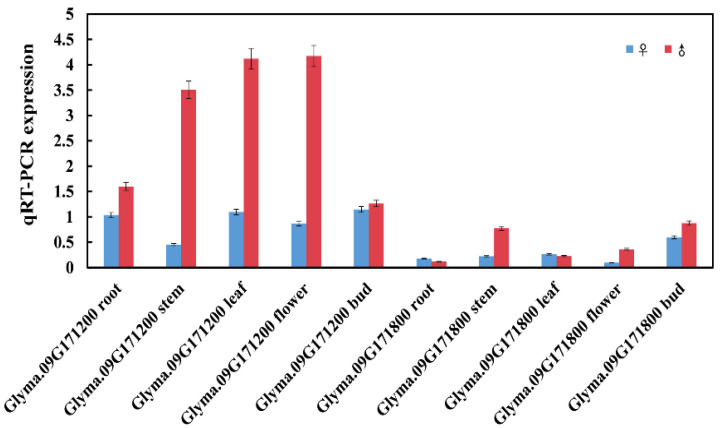
Gene expression analysis of *Glyma.09G171200* and *Glyma.09G171800* by qRT-PCR.

To verify whether *Glyma.09G171200* and *Glyma.09G171800* were the candidate genes for *Rf3*, qRT-PCR analysis was performed for analyzing the transcript levels of these two genes in five different organs of soybean, including root, stem, leaf, flower, and bud of the restorer and CMS lines (Figure 6). The expression levels of *Glyma.09G171200* at all stages were significantly higher in the restorer line than in the CMS line, especially in the stem, leaf, and flower organs. However, the transcript levels of *Glyma.09G171800* at stem, flower, and bud stages were slightly upregulated in the restorer line compared to the CMS line and downregulated in the root and leaf stages of the restorer line. Moreover, the gene expression level of *Glyma.09G171200* was significantly higher than *Glyma.09G171800* in all detective organs. Based on these results, the expression of *Glyma.09G171200* may better match the expression profile of the candidate gene.

Subsequently, ProtComp 9.0 was first used to predict the subcellular localization of the Glyma.09G171200 and Glyma.09G171800 protein. The results showed significant probability of mitochondrial localization of Glyma.09G171200 and extracellular (secreted) localization of Glyma.09G171800. Furthermore, SignalP—4.1 software predicted that only *Glyma.09G171200* encodes a protein containing pentatricopeptide repeats and had potential mitochondrial localization signals at its N-terminus. Since most reported *Rf* genes encode PPR proteins localized to the mitochondria, we propose *Glyma.09G171200* as the candidate gene for *Rf3*, and further subcellular localization analysis was performed.

**Figure 7:**
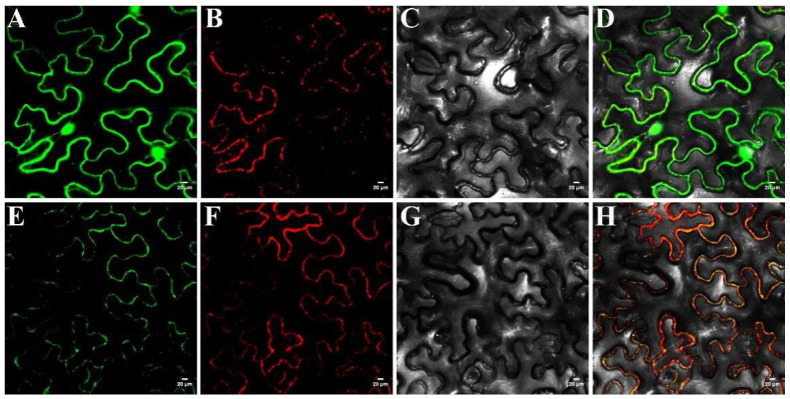
Subcellular localization of *Glyma.09G171200* in the mitochondria. (**A**) and (**E**) 35S: GFP and 35S: Rf3-GFP. (**B**) and (**F**) 35S: MSTP-mKate. (**C**) and (**G**) Bright field. (**D**) and (**H**) Merge of dark and bright field. Green shows the signal from GFP, red shows the signal from mKate. GFP, Rf3-GFP, and MSTP-mKate fusions under the control of the CaMV35S promoter were transiently expressed in tobacco protoplasts. Bar= 20 µm.

To further confirm subcellular localization of Glyma.09G171200, we expressed the GFP-fused Rf3 protein (green fluorescent protein) via a plasmid driven by the CaMV 35S promoter in tobacco protoplasts. The protein was targeted to the mitochondria based on the observation of the exclusively co-localized GFP signal with the MSTP-labeled mitochondrial signal (Figure 7). The fluorescence of MSTP and Rf3 seems to appear on the cell membrane or wall as mitochondria are squeezed to the edge of the cell wall by cytoplasmic inclusions, such as vacuoles. These results are in line with the ProtComp 9.0 prediction. Therefore, we propose *Glyma.09G171200* as the strongest candidate for *Rf3*.

### 2.7. Phylogenetic Analysis of the Rf3 Protein

**Figure 8:**
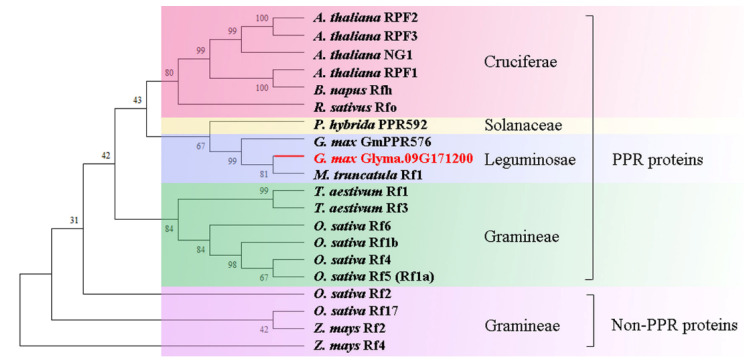
Phylogenetic analysis of Glyma.09G171200. The red branch indicates Glyma.09G171200. Bootstrap values of 1000 were used to assess the robustness of the neighbor-joining tree.

By searching the articles of *Rf* genes, we found 19 proteins (15 PPR and 4 non-PPR proteins) from different plants, including *Arabidopsis thaliana*, *Brassica napus*, *Petunia hybrida*, *Glycine max*, *Medicago truncatula, Triticum aestivum*, *Oryza sativa,* and *Zea mays*. To understand the evolutionary relationships of Glyma.09G171200 with other PPR proteins, we constructed a phylogenetic tree, including 20 proteins (including Glyma.09G171200). The phylogenetic analysis revealed that these proteins were mainly divided into four groups. The first group included six PPR proteins from cruciferous plants (*Arabidopsis thaliana*, *Raphanus sativus*, and *Brassica napus*). Notably, Glyma.09G171200 belonged to the second group. In this group, the genetic distance between Glyma.09G171200 and Rf1 of the leguminous plant *Medicago truncatula* was the smallest, and the genetic distance between Glyma.09G171200 and GmPPR576 of *Glycine max* was slightly farther. The third group included six PPR proteins from graminaceous crops (*Oryza sativa* and *Triticum aestivum*). The fourth group included the four non-PPR proteins (Figure 8). From these phylogenetic relationships, we concluded that the Glyma.09G171200 protein from JLR2 belongs to the PPR protein, clustered with the previously reported GmPPR576 protein from *Glycine max.* The result showed that *Glyma.09G171200* gene is consistent with most of the characteristics of *Rf* genes.

## 3. Discussion

The CMS/*Rf* system has been proven effective to produce hybrid seeds in crops. Considering the importance of these systems, numerous map-based cloning studies have been performed on *Rf* genes, and several genes have been isolated from various crops.

### 3.1. Fertility Restorer Type in JLR2 Is Controlled by an Uncharacterized New Gene

In soybean, recent research on *Rf* genes has mainly focused on CMS-RN, CMS-M, and CMS-N8855 types, among others. In the CMS-RN type, Wang et al. used 103 F_2_ individuals, derived from a cross between the CMS line JLCMS82A and the restorer line JIHUI 1, as the mapping population. The authors demonstrated that the mode of restoration was gametophytic and controlled by a single dominant locus (*Rf*). The genetic distances between the *Rf* gene and the SSR markers Sctt011 and Satt547 were 3.6 and 5.4 cm, respectively [24]. In the CMS-M type, Wang et al. suggested that *Rf-m* in the restorer line WR016 is a monogenic dominant gene located within a 162.4 Kb region on chromosome 16, which is flanked on each side by the markers GmSSR1602 and GmSSR1610, at a distance of 0.11 and 0.25 cM, respectively [26]. In the CMS-N8855 type, Yang et al. showed that two independent *Rf* loci from NJCMS1A are linked to Satt626 on chromosome 7 and Satt300 on chromosome 5 at the genetic distances of 9.75 and 11.18 cm, respectively [27]. Furthermore, Wang et al. suggested that the CMS line NJCMS1A is gametophyte sterility, and the fertility restoration of NJCMS1C to NJCMS1A is a quality trait controlled by a single gene locus. Moreover, *GmPPR576*, targeted to the mitochondria, was confirmed to be a fertility restorer gene in NJCMS1A, located on chromosome 16 between the markers BARCSOYSSR_16_1067 and BARCSOYSSR_16_1078 [22].

In the present study, we confirmed that JLCMS5A is gametophyte sterility, and the fertility restoration of JLCMS5A by JLR2 is a qualitative trait controlled by a pair of dominant fertility restoration genes, which we named *Rf3*. This result is consistent with the observations of Zhao et al. and Wang et al. that the mode of fertility restoration is gametophytic [23,24]. We constructed a genetic map of *Rf3* and noted that it is located within an 86.44 Kb region defined by the markers dCAPs09-2 and SSR_09_1170 on chromosome 9. The mapping interval of the *Rf3* gene locus is different from the previously reported loci [22,23,24,26,27], suggesting that the fertility restorer type in JLR2 is controlled by an uncharacterized novel gene.

### 3.2. Multiple PPR Genes and Fertility Restoration

In vascular plants, CMS, which is typically linked to an aberrant chimeric mitochondrial gene in the mitochondria, is conferred by genomic incompatibility between the mitochondria and nuclei [28]. In most cases, the male sterile phenotype can be recovered by a specific set of nuclear genes, termed the *Rf* genes, which encode RNA-binding PPR proteins [4,29,30]. PPR proteins, most of which are likely localized to the plastids and/or mitochondria, constitute a large nuclear-encoded RNA-binding protein family, which is further divided into P, PLS, and S subfamilies [31,32,33].

Numerous studies have indicated that the sterile phenotype of CMS lines can be restored by *Rf* genes, which encode PPR proteins. For instance, *Rf-PPR592*, the first *Rf* gene detected in petunia, encodes 14 PPR motifs [11]. The *Rfo* gene of radish encodes 16 PPR motifs [12]. *Rfp, Rfn*, and *Rfh* genes of rapeseed harbor PPR motifs [19,20,21]. In rice, *Rf1* encodes 16 PPR motifs [13]; while *Rf4*, *Rf5*, and *Rf6* encode typical P-type PPR motifs [1,15,16]. Similarly, *Rf1* and *Rf3* in wheat harbor PPR motifs [7].

In the present study, the mapping interval of the *Rf3* gene was 86.44 Kb. The annotation against the soybean Williams 82 reference genome revealed that this interval harbors 10 genes (Table 2) (*Glyma.09G171100*–*Glyma.09G172000*). Genomic sequence analysis revealed that two genes, *Glyma.09G171200* and *Glyma.09G171800*, exhibit SNPs that resulted in protein mutations in all *Rf3* genotype lines. However, only *Glyma.09G1712000* encodes a PPR protein. This result is consistent with previous reports on other major crops in which the *Rf* genes belong to the PPR family [1,7,11,12,13,15,16,19,20,21,22].

PPR family proteins are mainly involved in transcript processing in the chloroplasts and mitochondria [34]. All PPR-type *Rf* genes known thus far are targeted to the mitochondria [4]. By integrating ProtComp and subcellular localization analysis, we determined that *Glyma.09G1712000* is also localized to the mitochondria. Therefore, within the mapping interval, *Glyma.09G1712000*, which encodes a mitochondria-targeted PPR protein, is the strongest candidate gene for *Rf3*.

In conclusion, the F_2_ and F_3_ populations were selected as genetic populations to map *Rf3*. Using BSA-Seq analysis with SSR, InDel, and dCAPS markers, *Rf3* locus was identified in an 86.44 Kb candidate interval on chromosome 9. This region contains 10 putative genes. Among these, *Glyma.09G171200*, which encodes a mitochondria-targeted PPR protein, was proposed as the strongest candidate for *Rf3* based on sequence alignment and expression analysis. Finally, a CAPS marker co-segregated with *Rf3*, named CAPS1712, was developed. Although the present study proposes *Glyma.09G171200* as the restorer-of-fertility gene for JLCMS5A, the function of this gene warrants further exploration.

## 4. Materials and Methods

### 4.1. Plant Material

RN-type soybean CMS line JLCMS5A [S (*rf3rf3*)] was selected as the female parent, and it was crossed with the CMS restorer line JLR2 [N (*Rf3Rf3*)] in 2018. F_1_ plants [S (*Rf3rf3*)] were self-pollinated to create F_2_ mapping populations in 2019. In 2020, the CMS line, restorer line, and F_2_ seeds were cultivated for pollen sterility analysis and *Rf3* gene mapping at the experimental station of Jilin Academy of Agricultural Sciences, Fanjiatun, Changchun City, China (43°31′ N, 124°49′ E). All plant materials were provided by the Soybean Research Institute, Jilin Academy of Agricultural Sciences, Changchun, China. Cultivation practices, including soil preparation, fertilization, and irrigation, were consistent across all experiments.

### 4.2. Genetic Analysis

To investigate the inheritance of sterility, at least three blooming flowers from 217 F_2_ plants (from the cross of JLCMS5A with JLR2) were sampled. Anthers were dissected, squashed into an aqueous solution of 1% I_2_-KI, and observed under a microscope (Olympus, Japan). To determine the genetic model of *Rf3*, the observed values of fertile and semi-fertile plants were obtained, and the expected segregation ratio compatibility was assessed using the chi-square test in Excel.

### 4.3. Library Preparation and BSA-Seq

Total genomic DNA was extracted from the young leaves of soybean plants using the Plant Genomic DNA Kit (CWBIO, Jiangsu, China), according to the manufacturer’s instructions. The quality of the genomic DNA was monitored using 1% agarose gel electrophoresis. The concentration of each DNA sample was measured using the Nanodrop 2000 spectrophotometer (Thermo Fisher, Waltham, MA, USA) and adjusted to 1.5 μg DNA per sample. For BSA-Seq, four DNA bulks were prepared from male parents, female parents, fertile plants, and semi-fertile plants in the F_2_ population. The male and female parent bulks were constructed by mixing 10 μL of DNA each from 10 plants (labeled male and female), and the fertile and semi-fertile bulks were constructed by mixing 5 μL of DNA each from 20 plants exhibiting the fertile and semi-fertile phenotypes (labeled F and FS).

Libraries were prepared using the Truseq Nano DNA HT Sample Preparation Kit (Illumina, San Diego, CA, USA), following the manufacturer’s recommendations. Briefly, four DNA samples were fragmented by sonication to a size of 350 bp, and the DNA fragments were end-polished, A-tailed, and ligated with a full-length adapter for BSA-Seq, followed by PCR amplification. The PCR products were purified on the AMPure XP system. The libraries were analyzed for size distribution using the Agilent2100 Bioanalyzer and quantified using real-time PCR. Finally, the prepared libraries were sequenced using a paired-end read protocol, with 150 bp of data collected per run, on the Illumina Hiseq 4000 platform.

### 4.4. Sequencing Data Processing

To map the obtained sequences to the reference genome, clean reads were obtained from raw data by filtering the adaptor sequences, low-quality sequences, and unknown nucleotides (N), and stored in the FASTQ format [35]. The clean reads were mapped to the reference genomic sequence of Zhonghuang13 (https://ngdc.cncb.ac.cn/gwh/Assembly/125/show (accessed on 3 August 2020)) [36] using the Burrows–Wheeler aligner (BWA) (settings: mem -t 4 -k 32 -M -R) [37]. The alignment files were converted to the SAM/BAM format using SAMtools [38].

To identify and annotate the variants, reads at each position were filtered using the Genome Analysis Toolkit (GATK) package with the minimum base and mapping quality [39], and variant calling was performed for single-nucleotide polymorphisms (SNPs) and small InDels across bulks (settings: -Window 4, -filter “QD < 4.0|| FS > 60.0 || MQ < 40.0”, -G_filter “GQ < 20”). To determine the physical position of each variant, ANNOVAR [40] was used for aligning and annotating the SNPs or InDels.

To determine the candidate interval, sliding window analysis was applied to the frequency distribution of SNPs (SNP index) in the population of bulked individuals, and the SNP index was calculated for all SNP positions. SNP positions with an SNP index of <0.3 or >0.7 and the read depth of <7 from the two sequences were excluded, as these may represent spurious SNPs called due to sequencing and alignment errors. The Δ(SNP index) was calculated by subtracting the SNP indices of male and female bulk pools or the F and FS bulk pools. Quantitative trait loci (QTLs) were identified in the positive or negative peak regions with 95% confidence intervals in 10,000 bootstrap replicates. The selected SNPs and InDels in the peak regions were used to annotate and screen for the potential functional variants.

### 4.5. Marker Development and PCR Genotyping

To increase the marker density of the *Rf3* candidate region, various PCR-based markers, including SSRs, InDels, and SNPs, were developed based on the Zhonghuang13 genome [36] sequence and the BSA-Seq data. The PCR primer pairs showing polymorphisms between JLCMS5A and JLR2, as well as between fertile and semi-fertile F_2_ plants, were used to genotype 217 F_2_ plants (Table S3). For SSR analysis, PCR was performed in a 20 μL reaction mixture containing 50 ng DNA template, 1 μL each of forward and reverse primers (1 μM), and 12 μL of 2× Es Taq MasterMix (CWBIO, Jiangsu, China). The reaction conditions were as follows: initial cycle of 10 min at 95 °C, followed by 32 cycles of 30 s at 95 °C, 30 s at 50 °C, and 30 s at 72 °C, and a final cycle of 10 min at 72 °C. Denatured PCR products were separated on a 6% polyacrylamide gel in 1× TBE buffer and visualized using the silver staining method. Gel images were documented with a digital camera. For dCAPS analysis, the same PCR amplification protocol as that for SSR analysis was used, but the PCR products were digested with Xho I and the fragments separated on a 1% agarose gel. Based on the differences in genotypes as assessed using polymorphic markers, recombinants were identified in the 217 F_2_ plants and used to fine map *Rf3*.

### 4.6. Gene Cloning and Sequencing

The number and functional annotation of genes in the fine mapping interval were determined based on the soybean reference genome (https://phytozome-next.jgi.doe.gov/ (accessed on 10 July 2021)) and BSA-Seq data. For the above genes, PCR amplification was performed with primer pairs covering the full-length coding sequence (CDS) of each gene from the JLCMS5A and JLR2 lines, and genes with long sequences were divided into several segments for amplification with the corresponding number of primer pairs (Table S3). The PCR amplicons were purified and cloned using the pMD^TM^19-T Vector Cloning Kit (TaKaRa, Beijing, China). At least three randomly selected positive colonies for each amplicon were sequenced and assembled. Sequences of the above genes from the JLCMS5A and JLR2 lines were aligned using DNAMAN. For phylogenetic reconstruction, MEGA-X was used. The statistical method was maximum likelihood, and the phylogeny was examined using the bootstrap method with 1000 bootstrap values.

### 4.7. Expression Analysis

To validate their expression in the root, stem, leaf, flower, and bud of JLCMS5A and JLR2, the candidate genes were analyzed by real-time quantitative PCR (qRT-PCR). Total RNA was extracted using the RNApure Plant Kit (DNase I) (CWBIO, Jiangsu, China), and first-strand cDNA was synthesized using the SuperRT cDNA Synthesis Kit (CWBIO, Jiangsu, China) according to the manufacturer’s protocol. The housekeeping gene *Cons4* (GenBank accession number: BU578186) was used as the reference gene. All primer sequences used in the present study are listed in Table S3. qRT-PCR was performed in triplicate using the ChamQ Universal SYBR qPCR Master Mix (Vazyme, Nanjing, China) on the StepOnePlus Real-Time PCR System (Thermo Fisher, Waltham, MA, USA). Statistical analysis was performed using the well-known 2^−ΔΔCT^ method [41]. All data are expressed as mean ± standard deviation.

### 4.8. Subcellular Localization

ProtComp (http://www.softberry.com/ (accessed on 15 October 2021)) was used to predict the subcellular localization of Rf3. Further, a transient expression system was used to study the subcellular localization of the Rf3 protein. Briefly, the *Rf3* coding sequence, which lacked the stop codon, was generated through PCR using linker primers with the vector sequences ZF12 and ZR12 (Table S3). The subcellular localization vector pCAMBIA1302 was digested with Sal I and Pst I. The linker fragment was ligated to the linearized vector using T4 DNA Ligase (TaKaRa, Beijing, China) to construct the subcellular localization vector pCAMBIA1302: Rf3-GFP and pCAMBIA1302: MSTP-mKate for expression in *Nicotiana tabacum* L. protoplasts. The vector pCAMBIA1302-GFP was used as the control. The vector pCAMBIA1302: MSTP-mkate was used as mitochondrial marker. MSTP is a mitochondrial localization signal protein, whose amino acid sequence is MANRFRSGISFFKTIAVTDSVSSVRSKSLFPALRTYATASAQT [42]. The three vectors were injected into the abaxial surface of tobacco leaves, and the leaves were cultured for 72 h. Tobaccos were grown in pots containing a 1:1 mixture of forest vermiculite in a light chamber (400 μmol/m^2^/s) under 24 °C (day)/16 h and 3 °C (night)/8 h condition with 70% humidity. The images were obtained and observed under a laser confocal microscope (Nikon C2-ER, Japan). The excitation wavelengths for the green fluorescent protein (GFP) and far-red fluorescent protein (mKate) were 488 and 561 nm, respectively.

## Figures and Tables

**Table 1:** Quality appraisal of filtered reads in four samples.

Sample	Raw Base (bp)	Clean Base (bp)	Q20 (%)	Q30 (%)	GC Content (%)	Mapping Rate (%)	Average Depth (×)	Coverage at Least 1× (%)
Male	12,707,411,100	12,707,252,100	96.59	91.16	36.80	99.18	11.00	95.84
Female	10,235,684,400	10,205,530,500	97.39	92.68	37.99	99.55	8.64	93.00
F	31,164,198,000	31,090,225,200	97.72	93.54	36.14	99.60	24.12	97.99
SF	30,990,047,400	30,918,562,800	97.18	92.22	36.10	99.58	24.59	98.06
Mean	21,274,335,225	21,230,392,650	97.22	92.40	36.76	99.48	17.09	96.22
Sum	85,097,340,900	84,921,570,600	-	-	-	-	-	-

Note: Male, DNA pool of male parents; Female, DNA pool of female parents; F, DNA pool of fertile plants in F_2_; SF, DNA pool of semi-fertile plants in F_2_.

**Table 2:** Predicted genes and their information in the fine mapping region of *Rf3.*

Gene Name	Coding Region in the Longest Transcript	Locus	Description
*Glyma.09G171100*	1239 bp	39 607 686 bp~39 618 169 bp:+	Homeodomain-like superfamily protein
*Glyma.09G171200*	1494 bp	39 623 177 bp~39 625 286 bp:−	ATP binding; nucleic acid binding; helicases; pentatricopeptide repeat
*Glyma.09G171300*	90 bp	39 626 144 bp~39 626 702 bp:+	Cytochrome b6-f complex, subunit 8
*Glyma.09G171400*	105 bp	39 627 087 bp~39 627 482 bp: −	Photosystem II reaction center protein M
*Glyma.09G171500*	357 bp	39 630 511 bp~39 630 867 bp: −	ATP synthase subunit alpha
*Glyma.09G171600*	954 bp	39 644 737 bp~39 649 123 bp: −	Annexin 5
*Glyma.09G171700*	1344 bp	39 651 499 bp~39 653 374 bp:+	Mitochondrial transcription termination factor family protein
*Glyma.09G171800*	528 bp	39 653 896 bp~39 655 287 bp:+	Alpha/beta-Hydrolases superfamily protein
*Glyma.09G171900*	1245 bp	39 656 849 bp~39 660 683 bp: −	Protein of unknown function (DUF760)
*Glyma.09G172000*	2691 bp	39 664 672 bp~39 673 958 bp:+	Extra-large GTP-binding protein 3

Note: +/−, forward/reverse strand.

## Data Availability

Not applicable.

## Supplementary Materials

The following supporting information can be downloaded at: https://www.mdpi.com/article/10.3390/ijms23105388/s1.
