# Supplementary material for: Identification of a Candidate restorer-of-fertility Gene Rf3 Encoding a Pentatricopeptide Repeat Protein for the Cytoplasmic Male Sterility in Soybean
Source: Int J Mol Sci. 2022 May 11;23(10):5388. doi: 10.3390/ijms23105388 (PMC9140608; doi:10.3390/ijms23105388)
Supplement: Supplementary file 1 [file ijms-23-05388-s001.zip › ijms-1706266-supplementary/Supplementary/Table S1.pdf]

Table S1 SNPs of *Glyma.09G171200* DNA sequences

**Location**	**69**	**130**	**136**	**185**	**199**	**205**	**221**	**230**	**231**	**249**	**251**	**252**	**258**	**314**	**336**	**366**
Williams 82	G	G	A	C	G	G	G	C	G	G	A	T	G	A	A	C
Female	C	A	T	A	T	A	T	A	T	C	C	A	C	C	G	A
Male	G	G	A	C	G	G	G	C	G	G	A	T	G	A	A	C
**Location**	**381**	**411**	**445**	**447**	**461**	**478**	**529**	**582**	**591**	**629**	**630**	**655**	**671**	**672**	**691**	**766**
Williams 82	A	T	G	T	A	A	G	T	T	A	A	A	A	T	T	A
Female	G	G	A	C	G	C	A	C	C	T	G	G	C	C	G	T
Male	A	T	G	T	A	A	G	T	T	A	A	A	A	T	T	A
**Location**	**776**	**796**	**908**	**970**	**971**	**974**	**978**	**985**	**1119**	**1334**	**1395**	**1396**	**1407**	**1409**	**1446**	
Williams 82	G	T	A	G	G	T	G	A	T	T	A	T	A	C	T	
Female	A	C	G	A	A	G	C	G	C	A	T	G	C	T	C	
Male	G	T	A	G	G	T	G	A	T	T	A	T	A	C	T	
