# Supplementary material for: Identification of a Candidate restorer-of-fertility Gene Rf3 Encoding a Pentatricopeptide Repeat Protein for the Cytoplasmic Male Sterility in Soybean
Source: Int J Mol Sci. 2022 May 11;23(10):5388. doi: 10.3390/ijms23105388 (PMC9140608; doi:10.3390/ijms23105388)
Supplement: Supplementary file 1 [file ijms-23-05388-s001.zip › ijms-1706266-supplementary/Supplementary/Table S2.pdf]

Table S2 SNPs of Glyma.09G171200 protein sequences

**Location**	**23**	**44**	**46**	**62**	**67**	**69**	**74**	**77**	**83**	**84**	**105**	**137**	**149**	**154**	**160**	**176**
Williams 82	M	D	I	T	V	A	R	P	L	N	K	F	D	N	M	A
Female	I	N	F	N	L	T	M	H	F	T	T	L	N	S	L	T
Male	M	D	I	T	V	A	R	P	L	N	K	F	D	N	M	A
**Location**	**210**	**219**	**224**	**231**	**256**	**259**	**266**	**303**	**324**	**325**	**329**	**445**	**466**	**469**	**470**	
Williams 82	E	N	N	C	I	S	F	N	G	I	S	L	F	E	T	
Female	V	D	T	G	F	N	L	S	N	R	G	Q	V	D	I	
Male	E	N	N	C	I	S	F	N	G	I	S	L	F	E	T	
