# Supplementary material for: Identification of a Candidate restorer-of-fertility Gene Rf3 Encoding a Pentatricopeptide Repeat Protein for the Cytoplasmic Male Sterility in Soybean
Source: Int J Mol Sci. 2022 May 11;23(10):5388. doi: 10.3390/ijms23105388 (PMC9140608; doi:10.3390/ijms23105388)
Supplement: Supplementary file 1 [file ijms-23-05388-s001.zip › ijms-1706266-supplementary/Supplementary/Table S3.pdf]

Table S3 Primers used in this study

| Primer name | Sequence 5'-3' | Notes |
|---|---|---|
| SSR_09_1119F | GGCAGGACCAAATTGTGTTT | Fine mapping |
| SSR_09_1119R | TCAATGTGAATGGATCAGGTT | |
| SSR_09_1159F | AAAGAAGACGTGGGCAACAT | |
| SSR_09_1159R | ATGCGGAGTGATTTGGAGAG | |
| SSR_09_1161F | TCCCCATTATTTCACCATTTTC | |
| SSR_09_1161R | TGAACGATTTTACATCCCAAAA | |
| InDel09-3F | TTTGCATGAAACAGTGTTAAGGAG | |
| InDel09-3R | ATTAGGGTGAAAGGCGAGCGAGGG | |
| dCAPs09-2F | TAATCTATTTATTTTTTCTCTTCTT | |
| dCAPs09-2R | GTATTTTTTGACTATATTGTCTCGA | |
| SSR_09_1170F | CGTCTAAGGTTTTAGTTGGATTCTTT | |
| SSR_09_1170R | ACGCTCGCATGCATTTACTA | |
| SSR_09_1172F | CCTATTTTGGACCAGCCTCA | |
| SSR_09_1172R | AGTGCGGTTCCGTATTTGTT | |
| SSR_09_1178F | GCTTGAGGTGGCCAAATGTA | |
| SSR_09_1178R | GGATTGGTGACCATATTCATTG | |
| SSR_09_1184F | ACCTGCAGTCCTGCACATTT | |
| SSR_09_1184R | TGTCTGAGTCTATCCGCATTT | |
| SSR_09_1200F | ATCCACCCACAGTTACCCAA | |
| SSR_09_1200R | ATGGGGTGGAAACATTTGAA | |
| 11F5-1 | GTAACTAAAAATGCCCCAGA | Gene cloning |
| 11R5-1 | TTAACCCATAGATTGCTTGA | |
| 11F5-2 | CGCTAGAGGAGCACGGATAC | |
| 11R5-2 | TTAGGCATGTTCACTGTTGT | |
| 1712F2 | CGAGTCAGTAGAGGGATA | |
| 1712R2 | GACAGTAATCATTTGGCTAT | |
| 1713F3 | GGAGATAGGAGACAAAATTTAC | |
| 1713R3 | CTCAATTTTGAGTATCCCTA | |
| 1714F3 | GTTGAATTAAGTATCGATGAA | |
| 1714R3 | AATTAAGTCAGATTTCAGTG | |
| 1715F3 | ATGGTAACCATTCGTGCAGGTGAAA | |
| 1715R3 | TCAACCATCAATTGGTTTAGCCAGG | |
| 1716F3 | GGTGGACCAAGAAAGACTTAGT | |
| 1716R3 | AATCTTCATCTGTGCTATAGGC | |
| 1717F3 | ATGGCTTATGATATGTCCATAAGC | |
| 1717R3 | CTAACCATTTTGCATTTTCTTC | |
| 1718F12 | CAGAATAGCCCTGGTTACAG | |
| 1718R12 | AAAAGAAGGCAGGTACCAAT | |
| 19F5-1 | TAGCTGCCAATGGAAGCGGC | |
| 19R5-1 | AATGGTACCTTGAGTCGACG | |
| 20F6-1 | ATGGCGTCGGAATCCACCGGAGACG | |
| 20R6-1 | GATTACCTTTAACATTCTAAGCTC | |

| | | |
|---|---|---|
| 20F4-2 | AAGGACCTTTAAGTAGACAG | |
| 20R4-2 | AATTAAGATGGAAGCCAC | |
| 20F4-3 | TCGCAATCTTATGTTGTATAACTCC | |
| 20R4-3 | AGGGCTATCCAAGGTTGGGAAT | |
| Cons4-F | GATCAGCAATTATGCACAACG | qRT-PCR |
| Cons4-R | CCGCCACCATTCAGATTATGT | |
| Q11F1 | TAATAATGGTGTTGTTGGGGGT | |
| Q11R1 | ATTTGAAGAAAAATCAGAAGCT | |
| Q12F1 | GCATTATGTAAAGAAGGAAAGG | |
| Q12R1 | GTAAGTGTGAACGTCAGGAGTC | |
| Q13F1 | GGATATAATAAGTCTTGCTTGG | |
| Q13R1 | CTATAGTCCACTTCTTCCCCAT | |
| Q14F1 | ATTTATTGCTATTGCACTGTTT | |
| Q14R1 | AGTCACTTTGACTTACCGTTTT | |
| Q15F1 | ATTGCTCGTATTTATGGTCTTG | |
| Q15R1 | CTTTTACTGAACTTCCCTCTTG | |
| Q16F1 | GATCTATCATTCTATGTTTGGT | |
| Q16R1 | ATGTTTCATTCTTTACTGCCTT | |
| Q17F1 | TCTAGTGGATGTAGTGGAAAAC | |
| Q17R1 | TGCTGAGGATAGTGGGAATGTG | |
| Q18F3 | GCGCCTATAGTTTCATTTTTGC | |
| Q18R3 | GCAATTAGAGCGTCTGGATCTC | |
| Q19F1 | TTCTCTGATACCCTCCTTAACT | |
| Q19R1 | CTGCATAGACCTGTCCTACTCT | |
| Q20F1 | GATCTTTTTGAGAGTCTGGTGA | |
| Q20R1 | ACATAGTAATATGCTTGGTGCG | |
| ZF12 | ACGCGTCGACATGCTGTGTATGCGTCATA | Subcellular |
| ZR12 | AAAACTGCAGCAACAAGCCTCTAGCAATCA | localization |
