# Supplementary figures and images for: Identification of a Candidate restorer-of-fertility Gene Rf3 Encoding a Pentatricopeptide Repeat Protein for the Cytoplasmic Male Sterility in Soybean
Source: Int J Mol Sci. 2022 May 11;23(10):5388. doi: 10.3390/ijms23105388 (PMC9140608; doi:10.3390/ijms23105388)

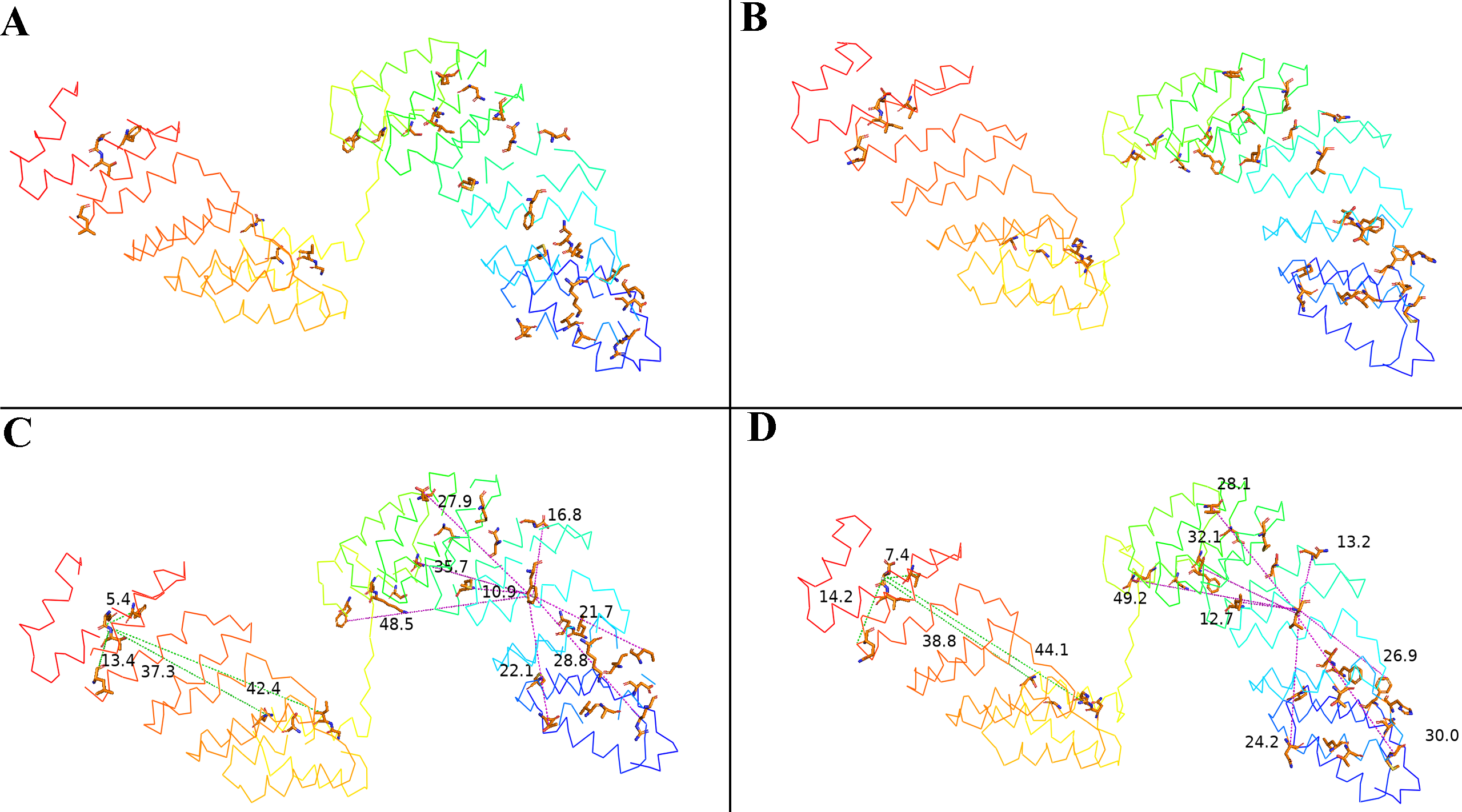

Supplement: Supplementary file 1 [file ijms-23-05388-s001.zip › ijms-1706266-supplementary/Supplementary/Figure S1.tif]

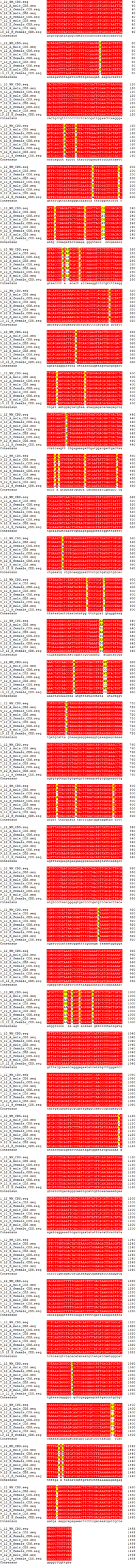

Supplement: Supplementary file 1 [file ijms-23-05388-s001.zip › ijms-1706266-supplementary/Supplementary/Figure S2.tif]

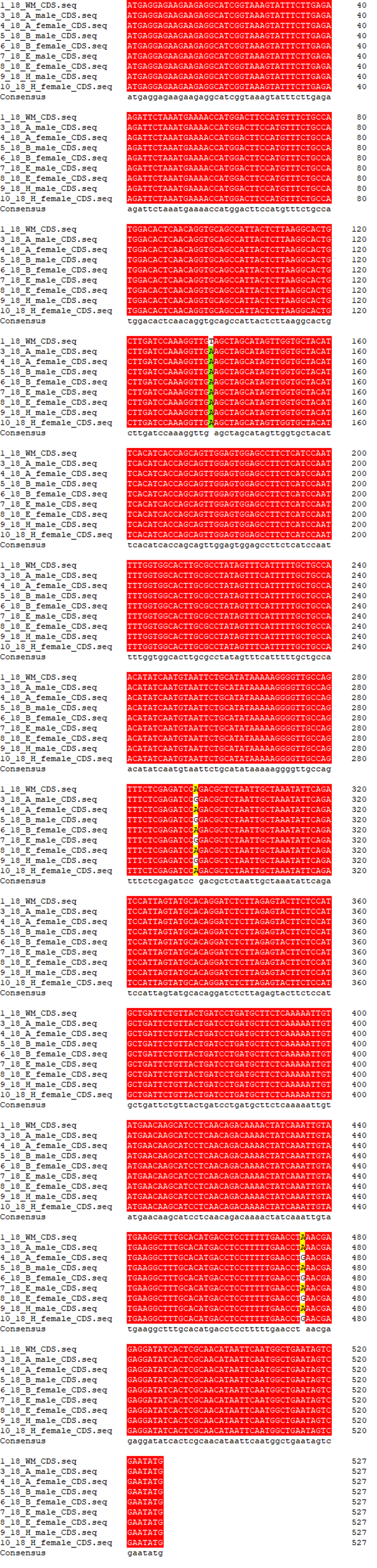

Supplement: Supplementary file 1 [file ijms-23-05388-s001.zip › ijms-1706266-supplementary/Supplementary/Figure S3.tif]
